# Feasibility and Impact of Left Atrial Appendage Closure in Patients with Cardiac Implantable Electronic Devices: Insights from a Prospective Registry

**DOI:** 10.3390/jcm14113857

**Published:** 2025-05-30

**Authors:** Tommaso Bini, Sven Ledwoch, Roberto Galea, Antanas Gasys, Marco Gamardella, George C. M. Siontis, Lorenz Räber, Laurent Roten

**Affiliations:** 1 Department of Cardiology, Bern University Hospital, University of Bern, 3010 Bern, Switzerlandmarco.gamardella@extern.insel.ch (M.G.);; 2 Graduate School for Health Sciences, University of Bern, 3012 Bern, Switzerland

**Keywords:** stroke prevention, atrial fibrillation, oral anticoagulation, pacemaker, implantable cardioverter-defibrillator

## Abstract

**Background**—Percutaneous left atrial appendage (LAA) closure (LAAC) offers a valid alternative to oral anticoagulation in patients with atrial fibrillation (AF) at high risk of bleeding. However, its impact on AF burden and device function in patients with cardiac implantable electronic devices (CIEDs) remains largely unexplored. **Methods**—From our prospective LAAC registry (clinicaltrial.gov—NCT04628078), which includes all consecutive LAAC procedures performed at our institution, we identified patients with a CIED and retrospectively analyzed procedural and follow-up data. The primary endpoint was defined as a composite of death, TIA/stroke, systemic or pulmonary embolism and major bleeding (BARC 3-5) within 7 days of the procedure. The secondary endpoint was CIED lead dislodgement. Additionally, AF burden was compared before and after LAAC. **Results**—Of the 586 LAAC procedures performed between August 2015 and January 2023, 36 patients (6%) had a CIED. The median CHA_2_DS_2_-VASC and HAS-BLED scores were 4.0 and 3.0, respectively. The primary endpoint occurred in one (3%) patient, and no patient experienced CIED lead dislodgement. AF burden data before and after LAAC were available in 20 patients. The mean AF burden increased from 6% to 31% following LAAC (*p* = 0.064). **Conclusions**—A CIED was present in 6% of LAAC procedures, and LAAC appears feasible and safe in this patient population. Larger, prospective studies are warranted to further evaluate the impact of LAAC on AF burden.

## 1. Introduction

Atrial fibrillation (AF) is the most common arrhythmia encountered in clinical practice and is associated with an increased risk of thromboembolic events [1]. Both European and American societies of cardiology assign a Class I Recommendation with a Level of Evidence “A” for the initiation of oral anticoagulation therapy (OAC) in patients with AF and elevated stroke risk to prevent thromboembolic events [2,3]. However, OAC is associated with an increased risk of bleeding, and its use may not be possible in all patients. The percutaneous closure of the left atrial appendage (LAA), the primary source of cardiac thrombi, has proven to be a feasible and valid stroke prevention strategy in patients who are unsuitable for or intolerant to long-term OAC, such as those at high bleeding risk [4].

In addition to its thrombogenic potential, the LAA may also serve as a trigger site for AF [5]. While pulmonary vein isolation (PVI) remains the cornerstone of catheter ablation for AF, some studies have demonstrated that LAA electrical isolation (LAAEI) can reduce AF recurrence in patients with LAA-triggered arrhythmia and in patients who do not respond to PVI [5,6,7,8,9]. The surgical exclusion or ligation of the LAA was also found to be effective in achieving LAAEI in various studies [10,11,12]. Afzal et al. investigated the impact of LAA closure (LAAC) using the LARIAT device in patients with cardiac implantable electronic devices (CIEDs) and reported a significant reduction in AF burden post-LAAC, suggesting that tissue necrosis around the LAA ostium may lead to LAAEI and subsequent AF burden reduction [12]. Comparable studies in patients undergoing transcatheter LAAC are lacking. Moreover, the introduction of large sheaths into the right atrium and subsequent transseptal puncture required for LAAC may pose a risk for lead dislodgement and potentially compromise CIED function. 

## 2. Methods

Since August 2015, all consecutive patients aged > 18 years undergoing LAAC at our institution are included in a prospective registry (ClinicalTrials.gov Identifier: NCT04628078) following written informed consent. From this registry, all patients with a CIED at the time of LAAC were identified up to the end of 2022. Data on patient clinical and procedural characteristics were collected prospectively. The study complies with the Declaration of Helsinki and the protocol was approved by the Ethics Committee of Bern (2016-01742) on 21 January 2021. Informed consent for participation was obtained from all subjects involved in the study.

LAAC procedure

The LAAC procedure has been described previously and was performed according to the local standard of care. Briefly, all procedures were carried out either under general anesthesia or deep sedation. Following transfemoral venous access, transseptal puncture was guided by transesophageal echocardiography (TEE) or under fluoroscopic guidance only. Particular care was taken to avoid the dislodgement of CIED leads during sheath pull-down from the vena cava superior and during subsequent transseptal puncture. After transseptal access was obtained, a device-specific sheath was advanced into the left atrium. LAAC was performed according to the instructions for use (IFU) of the respective device. The choice of occluder and of post-procedural antithrombotic therapy was at the discretion of the treating physician.

### 2.1. Follow-Up

At one year post procedure, clinical follow-up was systematically performed via telephone interviews. As per standard of care, a TEE follow-up was performed between 45 and 180 days after the procedure to assess for device-related thrombus (DRT) and peri-device leak (PDL). A PDL bigger than 4 mm were considered clinically significant. All clinical events were adjudicated by a committee consisting of two cardiologists, with a third cardiologist consulted in case of disagreement. Data on AF burden were retrospectively collected from regular CIED interrogations and reported when at least two device interrogations—one before to and one after the LAAC procedure—were available.

### 2.2. Study Endpoints

The primary endpoint was a composite of death, cerebrovascular accidents (including both stroke and transient ischemic attack [TIA]), systemic or pulmonary embolism and major bleeding (BARC 3–5) within 7 days of the procedure.

For the secondary endpoint the AF burden before and after the procedure was compared in patients with an active atrial lead.

### 2.3. Statistical Analysis 

Continuous variables are presented as median with interquartile range (IQR) and categorical variables as frequencies and percentages. The AF burden before and after the procedure was expressed as mean ± standard deviation and compared using the Wilcoxon test. A *p* value < 0.05 was considered statistically relevant. All statistical analysis were performed using IBM SPSS Statistics version 28.0.1.1 (SPSS Inc., Chicago, IL, USA).

## 3. Results

**Figure 1:**
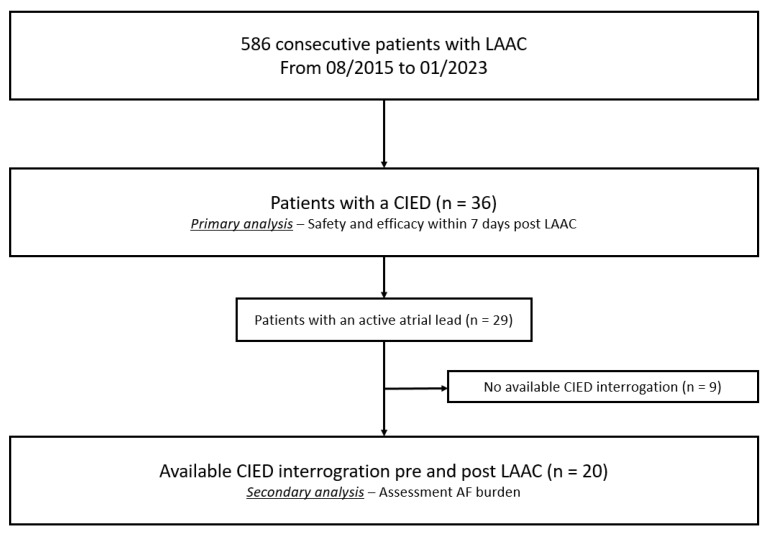
Study flowchart. LAAC = left atrial appendage closure; CIED = cardiac implantable electronic device.

Of the 586 consecutive LAAC procedures, 36 patients (6.1%) had a CIED (Figure 1). Baseline patient characteristics are summarized in Table 1. Median patient age was 73.5 years, and the majority were males (n = 27, 75%). The median CHA_2_DS_2_-VASc and HAS-BLED scores were 4.0 and 3.0, respectively. A pacemaker was implanted in 25 patients (69.4%) and an ICD in 11 (30.6%). An atrial lead was present in 29 patients (80.6%) and a ventricular lead in 39 patients. Of these, 6 had also a left ventricular lead. Active fixation was observed in 65 (88%) of the 74 leads, while the remaining 9 leads (3 in the right ventricle and 6 in left ventricle) were implanted with a passive fixation.

Procedural characteristics are reported in Table 2. Median procedure time was 36.5 min and no procedure was aborted. A disc–lobe type and a plug-based LAA occluder were implanted at equal proportions of the patient population (both n = 18; 50%).

The primary endpoint occurred in one patient (3%) and consisted of a major bleeding event (BARC 3a) localized at the knee occurring after knee arthroscopy, which required a transfusion of packed red blood cells. 

**Figure 2:**
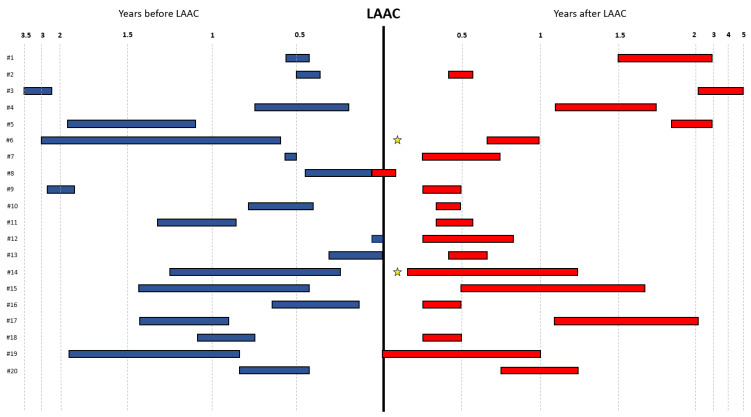
Timing of CIED interrogation with corresponding duration of atrial rhythm monitoring. CIED = cardiac implantable electronic device; LAAC = left atrial appendage closure; ☆ = electrical cardioversion.

**Figure 3:**
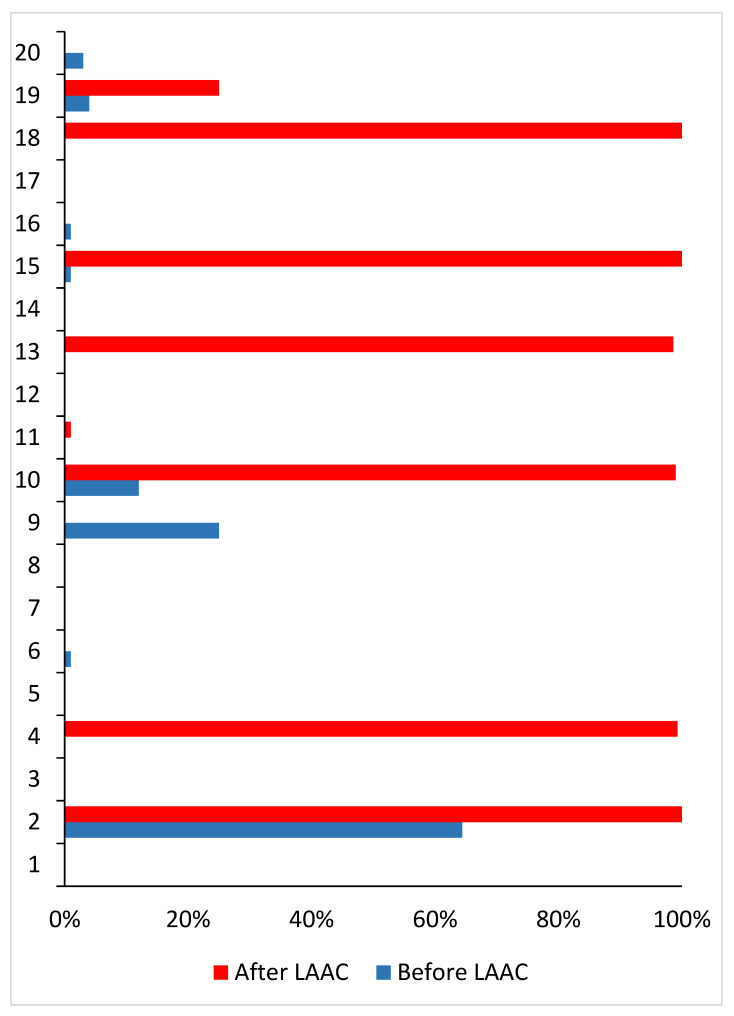
Individual AF burden before and after the LAAC procedure. AF = atrial fibrillation; LAAC = left atrial appendage closure.

Of the 29 patients with an active atrial lead, a CIED interrogation was available both prior to and following the LAAC procedure in twenty patients, of which four (20%) were dual-chamber ICDs, eleven (55%) dual-chamber pacemakers, one (5%) a CRT-P and four CRT-Ds (20%). The median number of days between pre-procedural CIED interrogation and LAAC, LAAC and post-procedural CIED interrogation and pre- and post-procedural CIED interrogation was 163.5 (IQR: 71.5; 300.5), 256.0 (IQR: 106.5; 389.5) and 416.0 (IQR: 340.5; 676.5), respectively. A rhythm control intervention with electrical cardioversion was performed in two patients (10%) after LAAC and before post-procedural CIED interrogation because of a ventricular tachycardia. The median durations of atrial rhythm monitoring before the corresponding CIED interrogations were 6.0 (IQR: 4:12) months for the pre-procedural CIED interrogation and 6.0 (IQR: 3; 12) months for the post-procedural CIED interrogation (Figure 2). The mean AF burdens during these time periods were 5.6% ± 15.1% for the pre-procedural CIED interrogation and 31.2% ± 46.2% for the post-procedural CIED interrogation LAAC (95% confidence interval −0.05–49.5; *p* = 0.064). Patient-level data of AF burden before and following LAAC are presented in Figure 3.

No lead dislodgement occurred during LAAC and no lead revision was necessary following LAAC.

Device-related complications at the TEE follow-up are reported in Table 3. At the median time 52.0 days (IQR: 48; 97), 31 patients (86%) underwent the echocardiographic follow-up. No DRT or significant PDL occurred. And only a minority of patients (n = 7, 22.6%) presented with a non-significant PDL.

The anti-arrhythmic drugs (AADs) before and after LAAC are reported in Table 4.

## 4. Discussion

The main findings of our study are as follows:

(1) A total of 6% of patients that underwent a LAAC procedure had a CIED, most commonly a pacemaker.

(2) LAAC in patients with a CIED is both feasible and safe, with no case of lead dislodgement observed.

(3) AF burden may increase following LAAC.

The presence of intracardiac leads may represent a hindrance during a cardiac procedure such as LAAC. In previous studies involving patients scheduled for catheter ablation, the overall rate of patients with CIED ranged between 5% and 10%, which is in line with our findings [13,14,15].

In our study, technical success was achieved in all cases, and the median procedure time was 36.5 min. This is comparable to procedure times reported in larger LAAC studies, suggesting that the presence of device leads does not affect procedural duration [16,17]. One patient experienced a procedure-related adverse event, namely a major bleeding. We observed no lead dislodgement, and no lead revision was necessary. However, CIEDs were implanted a median of 45.2 months (IQR: 13.2; 69.9) prior to LAAC and only a minority of patients had LV leads. Lead dislodgment may be more likely with recently implanted leads and passive fixation leads positioned in a cardiac vein. Overall, our findings are reassuring and suggest that percutaneous LAAC can be performed safely even in the presence of multiple device leads. Similar procedural safety outcomes have been reported in other studies using the same transfemoral venous access, but for different interventions such as transcatheter tricuspid or mitral edge-to-edge repair and catheter ablation, suggesting that this approach is safe in patients with CIEDs [13,18,19,20].

LAA ligation, through tissue compression and subsequent necrosis, appears to induce both the electrical and mechanical exclusion of the LAA [10,11,12]. Afzal et al. were the first to report the electrophysiological outcome of LAA ligation with the LARIAT device in patients with AF. In a cohort of 50 patients, they observed a significant reduction in AF burden at one year, particularly in patients with non-paroxysmal AF [12]. We found no reduction in AF burden in our study population. In contrast, AF burden at follow-up tended to be higher compared to values measured before LAAC. Importantly, the interval between pre- to post-procedural CIED interrogation was 416 days. Over such a time period, natural disease progression may occur, leading to an increased AF burden. The ATTEST trial, which included patients with paroxysmal AF, demonstrated that PVI can halt the progression from paroxysmal to persistent AF, whereas 17.5% of patients treated with antiarrhythmic drugs progressed to persistent AF within 3 years of follow-up [21]. Among patients with available AF burden measurements, we observed an AF burden of >95%—suggestive of persistent AF—in six (30%) patients after LAAC, compared to no cases before the procedure. Thus, the increased AF burden observed after LAAC was primarily driven by progression to persistent AF in these six patients. Notably, none of our patients underwent catheter ablation for AF.

Compared to LAAC with the LARIAT device or the surgical exclusion of the LAA, endocardial LAAC is not expected to result in sufficient tissue compression to induce LAA necrosis and subsequent reduction in AF burden. In the recently published OPTION trial, in which 803 patients were randomized to PVI versus PVI with additional LAAC, arrhythmia-free survival did not differ between groups, suggesting no effect of LAAC on AF burden [22]. Similarly, Romanov et al. randomized 96 patients to either PVI alone or PVI combined with LAAC and monitored AF burden over two years using an implantable cardiac monitor. In this study as well, no significant changes in AF burden were observed at two-year follow-up [23].

### Limitations

Our study has several limitations. First, this is a retrospective analysis with all inherent limitations associated with this study type. In particular, CIED interrogation was not uniformly scheduled and both the timing of CIED interrogation before and after LAAC, as well as the duration of atrial rhythm monitoring before CIED interrogation varied widely, rendering the estimation of AF burden imprecise. Second, no control group was available to compare the evolution of AF burden over time in the absence of LAAC. Third, the sample size was relatively small.

## 5. Conclusions

A CIED, most commonly a pacemaker, was present in 6% of patients undergoing a LAAC procedure. LAAC is both feasible and safe in patients with a CIED, and lead dislodgment is rare. Larger, prospective studies are warranted to further evaluate the impact of LAAC on AF burden over time.

## Figures and Tables

**Table 1:** Baseline patient characteristics.

	All Patients (n = 36)
Age (years), median ± IQR	73.5 (66; 78)
Sex, female n (%)	9 (25.0%)
Body mass index (kg/m^2^), median ± IQR	26.6 (24.1; 30.4)
Arterial hypertension, n (%)	29 (80.6%)
Diabetes mellitus, n (%)	14 (38.9%)
CHA_2_DS_2_-VASc score, median ± IQR	4.0 (3.0; 5.0)
HAS-BLED score, median ± IQR	3.0 (2.0; 3.0)
Paroxysmal AF, n (%)	22 (61.1%)
Persistent AF, n (%)	7 (19.4%)
Permanent AF, n (%)	3 (8.3%)
Unknown AF pattern, n (%)	1 (2.8%)
Atrial flutter, n (%)	3 (8.3%)
History of coronary artery disease, n (%)	17 (47.2%)
History of congestive heart failure, n (%)	22 (61.1%)
History of TIA or stroke, n (%)	6 (16.7%)
History of bleeding, n (%)	26 (72.2%)
History of kidney disease ^1^, n (%)	6 (16.7%)
History of liver disease ^2^, n (%)	0 (0.0%)
LVEF (%), median ± IQR	58.8 (37.5; 60.0)
LAVI (ml/m^2^), median ± IQR	44.6 (35.1; 54.2)
LA diameter (mm), median ± IQR	41.5 (37.8; 45.1)
Prior ECV, n (%)	9 (25%)
Prior PVI, n (%)	4 (11%)
**CIED**	
Single chamber ICD, n (%)	2 (5.5%)
Single chamber pacemaker, n (%)	5 (13.9%)
Dual chamber ICD, n (%)	4 (11.2%)
Dual chamber pacemaker, n (%)	19 (52.8%)
CRT-P, n (%)	1 (2.8%)
CRT-D, n (%)	5 (13.8%)

^1^ Defined as serum creatinine ≥ 200 umol/L, with the presence of chronic dialysis or prior renal transplantation. ^2^ Defined as chronic liver disease or biochemical evidence of significant hepatic dysfunction (bilirubin > 2x the upper limit of normal, in association with AST, ALT, or ALP at >3x the upper limit of normal). TIA = transient ischemic attack; LVEF = left ventricular ejection fraction; CIED = cardiac implantable electronic device; ICD = implantable cardioverter defibrillator; CRT-P = cardiac resynchronization therapy pacemaker; CRT-D = cardiac resynchronization therapy defibrillator; ECV = electrical cardioversion; PVI = pulmonary vein ablation.

**Table 2:** Procedural characteristics.

	All Patients (n = 36)
Procedure time (min), median (IQR)	36.5 (29.5; 45.0)
Fluoroscopy time (min), median (IQR)	11.7 (8.9; 15.7)
Contrast medium used (mL), median (IQR)	65.0 (46.0; 82.0)
General anesthesia, n (%)	4 (11%)
Conscious sedation, n (%)	32 (89%)
Fluoroscopic guidance	3 (8%)
TEE guidance, n (%)	33 (92%)
Diameter of LAA ostium by TEE (mm), median (IQR)	19 (18; 23)
Diameter of LZ by TEE (mm), median (IQR)	16 (14.5; 19)
LAA depth by TEE (mm), median (IQR)	21 (18; 24.5)
Chicken wing shape of LAA, n (%)	7 (19%)
Aborted procedure, n (%)	None
**LAAC-Device used**	
Watchman 2.5, n (%)	7 (19%)
Watchman FLX, n (%)	11 (31%)
Amulet/ACP, n (%)	18 (50%)
**Procedural complications within 7 days**	
Composite of death, cerebrovascular accident, systemic or pulmonary embolism and major bleeding, n (%)	1 (3%)
Death, n (%)	0 (0.0%)
Stroke or TIA, n (%)	0 (0.0%)
Systemic or pulmonary embolism, n (%)	0 (0.0%)
Major bleeding ^1^, n (%)	1 (3%)

^1^ defined as BARC Type 3-5. LAA = left atrial appendage; LZ = landing zone; TEE = transesophageal echocardiography; ACP = Amplatzer cardiac plug; TIA = transient ischemic attack.

**Table 3:** Device-related complications.

	All Patients (n = 36)
Days between LAAC and TEE follow-up, median (IQR)	52.0 (48; 97)
TEE follow-up, n (%)	31 (86%)
DRT, n (%)	None
Non-relevant PDL < 5 mm, n (%)	7 (22.6%)
Relevant PDL ≥ 5 mm, n (%)	None

LAAC = left atrial appendage closure; TEE = transesophageal echocardiography; DRT = device-related thrombus; PDL = peri-device leak.

**Table 4:** AAD before and after LAAC.

Before LAAC	All Patients (n = 36)
Beta blockade, n (%)	23 (63.9%)
CCA, n (%)	1 (2.8%)
Amiodarone, n (%)	8 (22.2%)
Digoxin, n (%)	1 (2.8%)
**After LAAC**	
Beta blockade, n (%)	30 (83.3%)
CCA, n (%)	1 (2.8%)
Amiodarone, n (%)	4 (11.1%)
Digoxin, n (%)	none

AAD = anti-arrhythmic drug; LAAC = left atrial appendage closure; CCA = calcium channel antagonist.

## Data Availability

The original contributions presented in the study are included in the article, further inquiries can be directed to the corresponding author.
